# Replacing up to 50% of Corn Silage with Triticale Silage Alters the Fecal Microbiome but Not Milk Yield or Composition in Mid-Lactation Holstein Cows

**DOI:** 10.3390/ani16071122

**Published:** 2026-04-07

**Authors:** Erlong Wang, Xiaoxia Han, Weidong Sun, Chen Zheng, Wenhua Du

**Affiliations:** 1 College of Animal Science and Technology, Gansu Agricultural University, Lanzhou 730070, China; wel1853103652@163.com (E.W.); hanxiaox0301@163.com (X.H.); 15583287738@163.com (W.S.); 2 Key Laboratory of Grassland Ecosystem, Gansu Agricultural University, Ministry of Education, Lanzhou 730070, China; duwh@gsau.edu.cn; 3 Pratacultural College, Gansu Agricultural University, Lanzhou 730070, China

**Keywords:** triticale silage, corn silage, lactating dairy cows, milk composition, fecal microbiota

## Abstract

Diversifying forage sources may improve the sustainability and flexibility of dairy production. In a 60 d feeding trial, 72 mid-lactation Holstein cows were assigned to three treatments (24 cows/group) and fed a total mixed ration in which corn silage represented 41.16% of dietary dry matter in the control diet; 25% or 50% of this corn silage fraction was replaced with triticale silage (TS) on a dry matter basis. The study evaluated whether partial TS substitution could maintain lactational performance while affecting fecal fermentation and microbiota. Replacing corn silage with TS did not affect milk yield, 4% fat-corrected milk, major milk components, or metabolic indicators. However, 50% replacement increased fecal bacterial richness and diversity, as reflected by ACE, Chao1, and Shannon indices, and altered the overall microbial community structure. This treatment also changed fecal volatile fatty acid profiles, including increasing the proportions of branched-chain volatile fatty acids. Overall, TS can replace up to 50% of the corn silage fraction in the ration of mid-lactation cows without compromising milk production or composition, while modifying hindgut microbial ecology and fermentation patterns, thereby offering greater ration flexibility when corn silage availability is limited or costly.

## Simple Summary

Under growing constraints in land use, climate variability, and feed costs, there is a need to diversify forage resources by identifying high-quality alternatives that can complement corn silage in dairy rations. This study evaluated triticale silage as a partial substitute for corn silage in mid-lactation Holstein cows under practical feeding conditions. Replacing up to 50% of the corn silage fraction with triticale silage did not alter milk yield, 4% fat-corrected milk, or milk composition. However, the 50% replacement increased fecal bacterial richness and diversity and shifted the overall microbial community structure. These findings suggest that triticale silage can serve as a complementary forage resource that maintains lactational performance while modulating the hindgut microbiota. From a farm-management perspective, this substitution strategy may increase ration flexibility when corn silage supply is limited or costly and supports forage diversification under variable climatic conditions.

## 1. Introduction

As large-scale dairy farming has expanded in China, the demand for high-quality roughage has continued to grow [1]. Currently, dairy cow diets rely heavily on corn silage (CS), which often constitutes a substantial proportion of the total mixed ration on a dry matter basis [2,3]. However, CS contains a large amount of readily fermentable starch [3], and feeding it at high proportions over the long term is likely to increase the likelihood of subacute ruminal acidosis (SARA) [4,5]. In addition, the variety of silage crops in China is rather limited. CS dominates, and this single-source roughage structure constrains the nutritional balance of rations [1]. Other silage crops such as ryegrass and alfalfa have relatively low cultivation area and yield, making it difficult to diversify the roughage portion of the diet [1]. Therefore, developing new roughage resources that are nutritionally complementary to CS is of great importance for safeguarding dairy cow health and achieving diversification of roughage supply.

In this context, triticale silage (TS) is increasingly considered a promising alternative forage for dairy systems [6,7]. Triticale originates from a cross between wheat and rye, combining the advantages of high yield, drought tolerance, strong stress tolerance, and broad adaptability [6]. Recent studies in other ruminant systems have also shown that alternative botanical feed resources can help sustain milk performance while modifying product quality, further underscoring the importance of forage diversification and sustainability-oriented ration design [8,9]. Silage made from triticale has a relatively high crude protein content and a moderate fiber structure [3,6,7], and it is rich in natural antioxidant compounds [10]. It can serve as a nutritional complement to CS in the diet and may influence the rumen and hindgut microbial environment [2,11,12,13], although its contributions under practical dairy-cow feeding conditions require further validation.

However, current studies on the use of TS in dairy production remain limited and not systematic [6,7]. In particular, there is a shortage of work evaluating how practical partial substitution of CS with TS affects milk responses together with hindgut fermentation and fecal microbiota under commercial feeding conditions. In the present study, 25% and 50% replacement levels were selected to represent moderate and upper-bound partial substitution scenarios that could be implemented on-farm while maintaining a stable TMR structure and avoiding full reformulation of the forage base [3,7]. Accordingly, this experiment was designed as a practical partial-replacement evaluation rather than a full replacement-value assay; it was not intended to determine the absolute nutritional value of TS or an optimal replacement ratio. Therefore, we evaluated the effects of replacing 0%, 25%, or 50% of the CS fraction with TS on milk yield, milk composition, fecal volatile fatty acids, and fecal microbiota of lactating dairy cows.

## 2. Materials and Methods

### 2.1. Study Location and Ethical Approval

This study was conducted from August to October 2025 at Hexie Animal Husbandry Science and Technology Co., Ltd. (Dingxi, China). and the Hexie Dairy Cattle Breeding Specialized Cooperative in Balipu Town, Lintao County, Gansu Province, China (mean altitude: 2055 m; 103°52′37.77″ E, 35°24′16.65″ N). The lactating dairy cows used in this trial were provided by the same farm. All procedures were conducted in accordance with the guidelines approved by the Ethics Committee of Gansu Agricultural University (approval no. GSAU-Eth-AST-2024-023).

### 2.2. Silage Management and Chemical Composition

Triticale silage (TS) was cultivated, harvested, and ensiled in Zhangjiazhuang Village, Balipu Town, Lintao County. It was prepared from the first cut harvested at the grain-filling stage and stored in a bunker silo for more than 30 d before use. Corn silage (CS) was cultivated after triticale harvest, harvested at the wax-ripeness stage, and ensiled in a bunker silo for more than 30 d before use. Both silages were stored in bunker silos for more than 30 d before use. Quantitative records for pH at silo opening, packing density, and exact ensiling duration beyond this general storage period were not archived and therefore are not reported here. Silage samples were collected weekly throughout the experiment and analyzed to characterize the silages offered. The chemical compositions of the silages are shown in Table 1.

### 2.3. Cows, Experimental Design, and Feeding Management

A single-factor completely randomized design was used. The control group (CON) received a basal TMR in which CS served as the primary silage source, whereas in the treatment groups, 25% (TS25) or 50% (TS50) of the CS fraction was replaced with TS on a dry matter basis. A total of 72 healthy mid-lactation Holstein cows were enrolled (average milk yield 30 ± 3.00 kg/d; body weight 600 ± 30.00 kg; days in milk 110 ± 10 d). Cows were first stratified by parity and then randomly allocated within parity strata to the three treatments so that each treatment included 5 primiparous and 19 multiparous cows. For milk yield, milk composition, fecal microbiota, and fecal VFA measurements, the individual cow was used as the statistical unit for analysis. Cows in each treatment were managed in a separate pen for feeding, resulting in one pen per treatment for feed delivery and refusal recording. Because the dietary treatments differed in silage source and were managed at pen level, blinding of farm staff during feeding and routine sampling was not feasible under the farm conditions. The study included a 15-d adaptation period followed by a 60-d experimental period. Cows were fed twice daily at 07:00 and 17:00 h and milked twice daily at 06:00 and 16:00 h. Except for differences in silage source, all other dietary components were identical among treatments. Diets were formulated to meet the nutritional requirements of cows producing 30 kg milk/d according to NRC (2001) [14]. The ingredient composition and nutrient levels of the experimental diets are presented in Table 2.

### 2.4. Sample Collection, Laboratory Analyses, and Measurements

#### 2.4.1. Silages and Total Mixed Diets

Silage, TMR, and ort samples were collected weekly throughout the 60-d experimental period. For each week, representative samples were composited by feed type, dried, ground, and analyzed separately; the table values reported for silages and diets represent the mean of these repeated analytical measurements over the feeding period. DM (method 930.15), Ash (method 942.05), CP (method 990.03), EE (method 920.39), Ca (method 927.02), and phosphorus (method 965.17) were determined according to AOAC (2002) [15]. NDF and ADF contents were measured following the method described by Van Soest et al. (1991) [16].

#### 2.4.2. Milk Yield, Milk Composition and Related Parameters

Milk yield was recorded daily for each cow. Milk samples were collected from each cow on day 30 and day 60. Because the interval before the morning milking (06:00 h) was approximately 14 h, whereas that before the evening milking (16:00 h) was approximately 10 h, morning and evening milk were pooled in a 6:4 ratio to obtain a 100 mL composite sample representative of daily milk output, which was thoroughly mixed (potassium dichromate was added in the sampling tube as a preservative). The milk samples were stored at 4 °C and promptly sent to the Lanzhou Institute of Animal Husbandry and Veterinary Medicine Dairy Herd Improvement (DHI) Testing Laboratory for analysis. The measured milk parameters included milk fat percentage (Fat), milk protein percentage (Protein), lactose percentage (Lactose), milk urea nitrogen (MUN), somatic cell count (SCC), milk total solids (Tots), milk acetone (Acetone) concentration, and β-hydroxybutyrate (BHBA) content.

The 4% fat-corrected milk (4% FCM) yield was calculated according to the equation of Gaines and Davidson (1923) [17]. Using the following equation:

4%FCM(kg)=M(kg)×(0.4+0.15×F)

where M is the milk yield and F is the Fat %.

#### 2.4.3. Fecal Microbiota

At the end of the trial (day 60), rectal fecal samples (~5 g each) were obtained from eight cows per treatment once daily over three consecutive days before the morning feeding. The three daily samples from each cow were pooled in equal proportions to obtain one composite sample per cow (*n* = 8 per treatment) for DNA extraction and microbial analysis. Samples were placed into sterile 5 mL cryotubes, rapidly snap-frozen in liquid nitrogen, and stored at −80 °C until further processing.

Microbial DNA was obtained with the E.Z.N.A.^®^ Soil DNA Kit (Omega Bio-tek, Norcross, GA, USA) following the manufacturer’s instructions. DNA integrity, concentration, and purity were assessed by agarose gel electrophoresis and NanoDrop 2000 spectrophotometry (Thermo Fisher Scientific, Waltham, MA, USA), respectively. The V3–V4 region of the 16S rRNA gene was PCR-amplified using barcoded primers 338F and 806R. Reactions (20 μL) contained TransStart FastPfu buffer, dNTPs, primers, FastPfu polymerase, and template DNA. Cycling parameters on an ABI 9700 thermal cycler were: 95 °C for 3 min; 27 cycles of 95 °C (30 s), 55 °C (30 s), 72 °C (30 s); and a final 72 °C for 10 min. For each sample, triplicate amplicons were pooled, verified by gel electrophoresis, and purified using the AxyPrep kit (Axygen Biosciences, Union City, CA, USA). Purified amplicons were quantified with the QuantiFluor^TM^-ST system (Promega, Madison, WI, USA) and pooled at equimolar ratios. Libraries were prepared using the TruSeq^TM^ DNA Sample Prep Kit (Illumina, San Diego, CA, USA) and sequenced on the Illumina NextSeq 2000 platform at Majorbio Bio-Pharm Technology Co., Ltd. (Shanghai, China).

The raw paired-end reads were processed using FASTP (v0.23.4) [18] for quality control. Sequences with a Phred quality score below 20 at either end were trimmed, and a sliding window of 50 bp was applied to trim reads with an average quality score below 20. Reads shorter than 50 bp or containing more than 5 ambiguous nucleotides (N) were discarded. High-quality reads were merged using FLASH (v1.2.11) [19] with a minimum overlap of 10 bp and a maximum mismatch ratio of 0.2. The merged reads were demultiplexed by sample, ensuring correct identification based on their corresponding barcodes and primers.

After quality control, sequences were grouped into operational taxonomic units (OTUs) at 97% identity using USEARCH (v11) [20,21]. Chimeric sequences were identified and removed during clustering to improve accuracy. Taxonomic assignment of representative sequences was done using the RDP Classifier (v2.11) [22] against the SILVA 138 16S rRNA reference database [23] with a confidence threshold of 70%. Sequences identified as chloroplast or mitochondrial DNA were discarded as potential contaminants. To account for differences in sequencing depth, the read counts for each sample were rarefied to the lowest sequencing depth (30,449 reads).

Alpha-diversity indices (Chao1, ACE, Shannon, and Simpson) and rarefaction curves were calculated using mothur (v1.48.3) [24] and visualized in R. Beta-diversity was assessed on Bray–Curtis dissimilarities and presented via principal coordinates analysis (PCoA). Differences in community structure between groups were evaluated with ANOSIM tests. Additionally, differentially abundant taxa between groups were identified using LEfSe [25] with a logarithmic LDA score cutoff of 3.5 for discriminative features. Spearman’s rank correlation was used to examine associations between milk performance traits and the relative abundances of dominant genera (top 20 genera by relative abundance), and the resulting correlation matrix (correlation coefficients and significance levels) was visualized as a heatmap. All bioinformatic analyses were performed using the Majorbio Cloud Platform (https://www.majorbio.com).

#### 2.4.4. Fecal Volatile Fatty Acids

An additional aliquot of the end-trial composite fecal sample collected from the same cows described above was used for VFA analysis. For VFA extraction, a supernatant was prepared from the fecal samples and centrifuged, after which an internal standard was added. Samples were vortexed, allowed to stand, and centrifuged again. The final supernatant was analyzed using a GC-4800A gas chromatograph (Beijing Dongxi Electronic Technology Co., Ltd., Beijing, China) equipped with a flame ionization detector. Volatile fatty acid concentrations and molar proportions were quantified and expressed relative to total volatile fatty acid (TVFA) concentration.

### 2.5. Statistical Analysis

Statistical analyses were performed using R software (Version 4.3.3). Repeated-measures mixed-effects models were fitted in R, and when an autoregressive covariance structure was specified, models were fitted using the nlme package. Daily milk yield data were analyzed using a linear mixed-effects model (LMM) that included the fixed effects of treatment group (CON, TS25, TS50), sampling day, and their interaction. Parity, days in milk (DIM), body weight (BW), and baseline milk yield were included as covariates. Cow identity was treated as a random intercept to account for repeated measurements. A first-order autoregressive (AR(1)) covariance structure was specified for within-cow residuals to model the correlation among repeated daily measurements. This AR(1) modeling was implemented using the nlme package to enable correlation structure specification. The model can be expressed as:

$$Y_{ijk}=\mu +G_{i}+T_{j}+{\left(G\times T\right)}_{ij}+\beta X_{k}+C_{k}+\epsilon_{ijk}$$

where *Y_ijk_* is the response variable (daily milk yield), μ is the overall mean, *G_i_* is the fixed effect of treatment (with levels CON, TS25, TS50), *T_j_* is the fixed effect of day *j*, (*G* × *T*)_*ij*_ is the treatment-by-day interaction, *X_k_* is the vector of covariates (parity, DIM, BW, and baseline milk yield) with regression coefficients *β*, *C_k_* is the random effect of cow *k* (assumed *C_k_*~N(0, *σ*_*C*^2^_)), and *ϵ_ijk_* is the residual error term. An autoregressive covariance structure of order 1 was used to model the within-cow residual correlations across days. Model assumptions (normality and homoscedasticity of residuals) were assessed using diagnostic plots, and potential outliers were screened. Fixed effects were evaluated using F-tests based on Type III sums of squares. Results are reported as least-squares means (LSMeans), with SEM denoting the pooled standard error of the LSMeans. Orthogonal polynomial contrasts were applied to test linear (L) and quadratic (Q) responses to increasing TS inclusion. 

Milk composition and quality measured at mid- and end-trial were analyzed using the same linear mixed-effects model described above. Traits measured once at end-trial (Lactose, Acetone, and BHBA) were analyzed by ANCOVA:


$$Y_{ik}=\mu +G_{i}+\beta X_{k}+\epsilon_{ik}$$

Somatic cell count was analyzed after log10 transformation to improve normality.

Statistical significance determined at *p* < 0.05 for all tests. For multiple comparisons, *p*-values were adjusted using the Benjamini–Hochberg false discovery rate (FDR) method, and FDR-adjusted *p*-values (*q*-values) less than 0.05 were considered significant.

## 3. Results

### 3.1. Effects of Partially Replacing Corn Silage with Triticale Silage on Milk Yield and 4% FCM

As shown in Table 3, partially replacing CS with TS did not affect milk yield or 4% FCM (*p* > 0.05). Numerically, mean milk yield ranged only from 29.41 to 29.78 kg/d across treatments, and 4% FCM ranged from 30.01 to 30.73 kg/d, indicating very small between-treatment differences under the present conditions. Both variables were significantly affected by sampling day (*p* < 0.001), and a significant treatment × day interaction was detected for 4% FCM (*p* = 0.020). Trend analysis showed that none of the parameters exhibited a significant linear and quadratic trend across the replacement levels (*p* > 0.05).

### 3.2. Effects of Partially Replacing Corn Silage with Triticale Silage on Milk Quality of Lactating Holstein Cows

According to Table 4, no overall treatment effects were observed for Fat, Protein, Tots, MUN, SCC, Lactose, Acetone, or BHBA (*p* > 0.05). Numerically, Fat ranged from 4.10 to 4.27%, Protein from 3.35 to 3.44%, and SCC from 9.86 to 16.89 × 10^3^ cells/mL across treatments, indicating only limited between-treatment differences. MUN and SCC remained within normal physiological ranges for all groups. Sampling time significantly affected milk protein percentage and MUN concentration (*p* < 0.05), whereas no significant treatment × time interactions were detected for any parameter (*p* > 0.05). Furthermore, no significant linear or quadratic responses were identified for any measured parameter across the replacement levels (*p* > 0.05).

### 3.3. Effects of Replacing Corn Silage with Triticale Silage on the Fecal Microbiota of Dairy Cows

#### 3.3.1. Sequencing Depth and OTU Distribution

**Figure 1:**
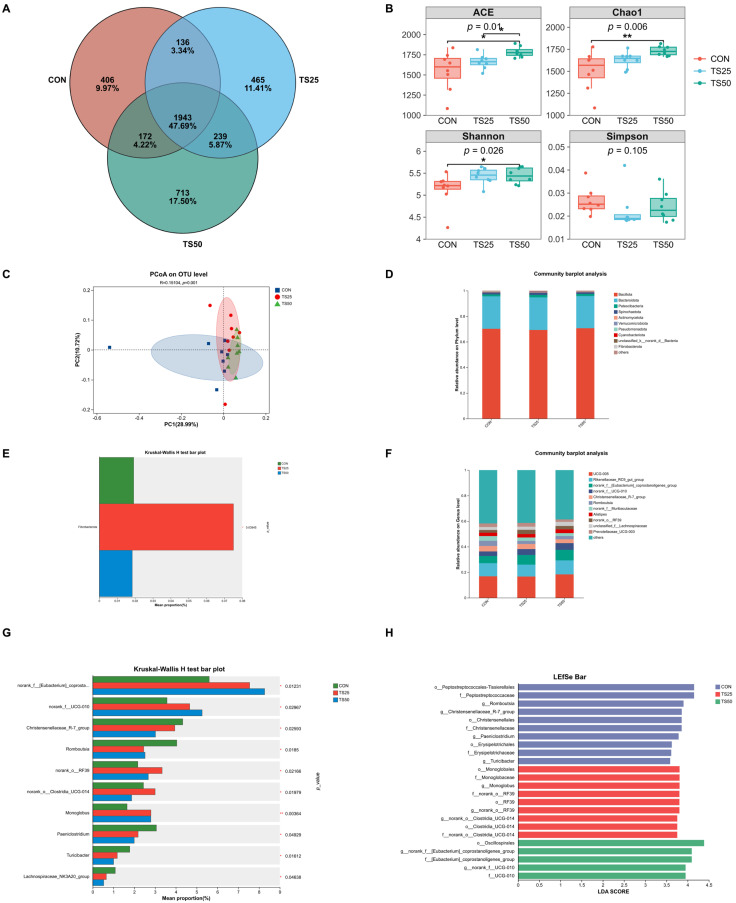
Effects of partially replacing corn silage with triticale silage on fecal bacterial community in lactating cows. (**A**) Venn diagram showing shared and unique OTUs among treatments. (**B**) Alpha-diversity indices (ACE, Chao1, Shannon, and Simpson). (**C**) Principal coordinates analysis (PCoA) based on Bray–Curtis dissimilarities at the OTU level. (**D**) Relative abundance of major bacterial phyla. (**E**) Differential relative abundance of Fibrobacterota (Kruskal–Wallis test). (**F**) Relative abundance of dominant bacterial genera. (**G**) Differential genera identified by Kruskal–Wallis tests. (**H**) LEfSe results (LDA score > 3.5). * *p* < 0.05, ** *p* < 0.01. CON, control. TS25, 25% replacement. TS50, 50% replacement.

Rarefaction curves plateaued with increasing sequencing depth, indicating sufficient coverage of fecal microbiota diversity. A total of 4074 operational taxonomic units (OTUs) were identified at 97% similarity, with 406 unique to the CON group, 465 unique to TS25, 713 unique to TS50, and 1943 shared by all three groups (Figure 1A).

#### 3.3.2. Alpha Diversity

Alpha diversity analysis showed that the TS50 group harbored a richer and more diverse microbiota compared to the other groups. Specifically, the TS50 group exhibited significantly higher Ace and Shannon indices than the CON group (*p* < 0.05), along with a markedly higher Chao1 richness index (*p* < 0.01). The ACE index in the TS50 group was significantly higher than in the TS25 group (*p* < 0.05) (Figure 1B).

#### 3.3.3. Beta Diversity

Principal coordinates analysis (PCoA) of beta diversity at the OTU level showed that PC1 and PC2 explained 28.99% and 10.72% of the total variation, respectively. The distribution of samples in the PCoA plot indicated distinct community structures among the three groups. ANOSIM analysis further confirmed that overall microbial community composition differed significantly between groups (R = 0.15104, *p* = 0.001). Notably, samples from the CON and TS50 groups formed clearly separated clusters in the PCoA space, suggesting the greatest divergence in their fecal microbiota. The TS25 group occupied an intermediate position, with some TS25 samples overlapping the CON group cluster and others shifting towards the TS50 group cluster (Figure 1C).

#### 3.3.4. Taxonomic Composition and Differential Relative Abundance

At the phylum level, most phyla did not differ significantly among the three groups (Figure 1D). The only exception was Fibrobacterota, which was significantly more abundant in the TS25 group than in both the CON and TS50 groups (*p* = 0.039), while other phyla did not differ significantly (*p* > 0.05) (Figure 1E).

At the genus level, the fecal microbiota was dominated by several taxa, including *UCG-005*, Rikenellaceae RC9 gut group, and *Eubacterium_coprostanoligenes_*group (Figure 1F). Notably, the *Eubacterium_coprostanoligenes_*group was present at significantly higher relative abundance in both the TS25 and TS50 groups compared to the CON group (*p* = 0.012), whereas the genus *Romboutsia* was significantly less abundant in TS25 and TS50 than in CON (*p* = 0.019). Additionally, the relative abundance of genus *UCG-010* differed significantly among the three groups (*p* = 0.030), and the Lachnospiraceae_NK3A20_group also showed a significant difference in relative abundance (*p* = 0.046) (Figure 1G).

Using linear discriminant analysis effect size (LEfSe) with an LDA score > 3.5, we further identified key bacterial taxa that distinguished the microbial communities of each group. The LEfSe results indicated that each group was characterized by enrichment of different signature taxa. The CON group was primarily enriched in *Romboutsia*, Christensenellaceae_R-7_ group, and *Turicibacter*. In the TS25 group, the taxa most enriched belonged to the order Monoglobales (family *Monoglobaceae*, genus *Monoglobus*), as well as members of the RF39 order and the Clostridia *UCG-014* clade. In contrast, the TS50 group was mainly enriched in the order *Oscillospirales*, the *Eubacterium_coprostanoligenes_*group, and taxa related to *UCG-010* (Figure 1H).

### 3.4. Fecal Volatile Fatty Acids

**Figure 2:**
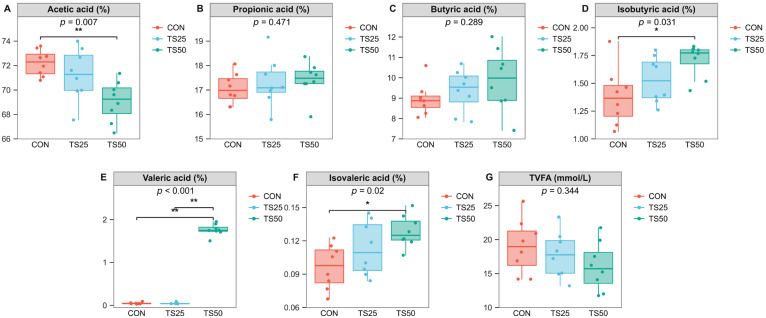
Effects of partially replacing corn silage with triticale silage on fecal volatile fatty acids in lactating cows. (**A**) Acetic acid proportion. (**B**) Propionic acid proportion. (**C**) Butyric acid proportion. (**D**) Isobutyric acid proportion. (**E**) Valeric acid proportion. (**F**) Isovaleric acid proportion. (**G**) Total volatile fatty acid (TVFA) concentration (mmol/L). * *p* < 0.05, ** *p* < 0.01. CON, control. TS25, 25% replacement. TS50, 50% replacement.

As shown in Figure 2, total volatile fatty acid (TVFA) concentration was not affected by dietary treatment (*p* = 0.344). The molar proportion of acetic acid was significantly lower in TS50 than in CON (*p* = 0.007), whereas the molar proportions of propionic acid and butyric acid did not differ among treatments (*p* = 0.471 and *p* = 0.289, respectively). In contrast, TS inclusion altered several minor VFA fractions. TS50 increased the molar proportions of isobutyric acid (*p* = 0.031) and isovaleric acid (*p* = 0.020) relative to CON. Valeric acid showed the most pronounced response, with TS50 exhibiting a markedly higher molar proportion than both CON and TS25 (*p* < 0.001).

### 3.5. Correlations Between Fecal Microbiota, Milk Traits, and Fecal Volatile Fatty Acids

**Figure 3:**
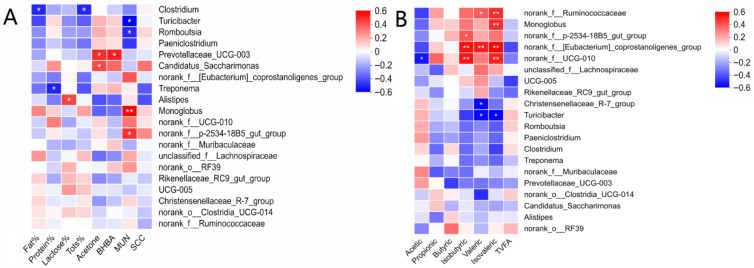
Spearman correlation heatmaps between fecal bacterial genera and milk traits or fecal volatile fatty acids in lactating cows. (**A**) Correlations between milk traits (Fat%, Protein%, Lactose%, Tots% (total solids), Acetone, BHBA (β-hydroxybutyrate), MUN (milk urea nitrogen), and SCC (somatic cell count)) and the top fecal genera. (**B**) Correlations between fecal volatile fatty acid profiles (Acetic, Propionic, Butyric, Isobutyric, Valeric, and Isovaleric acids and TVFA (total volatile fatty acids)) and the top fecal genera. Color intensity indicates Spearman’s correlation coefficient (red, positive; blue, negative). * *p* < 0.05, ** *p* < 0.01.

Spearman correlation analysis was performed between milk traits and the relative abundances of dominant fecal genera (top 20) (Figure 3A). *Monoglobus* and norank_f__p-2534-18B5_gut_group were positively correlated with MUN, whereas *Turicibacter* and *Romboutsia* were negatively correlated with MUN (*p* < 0.05). *Prevotellaceae_UCG-003* was positively correlated with both milk Acetone and BHBA, and Candidatus*_Saccharimonas* was positively correlated with milk Acetone (*p* < 0.05). *Clostridium* was negatively correlated with Fat and Tots, *Treponema* was negatively correlated with Protein, and *Alistipes* was positively correlated with Lactose (*p* < 0.05). Correlation analysis between fecal genera and fecal VFA molar proportions (Figure 3B) showed that significant associations were mainly observed for minor and branched-chain VFAs. norank_f__Ruminococcaceae was positively correlated with valeric and isovaleric acid proportions, *Monoglobus* was positively correlated with isovaleric acid, norank_f__p-2534-18B5_gut_group was positively correlated with isobutyric acid, and norank_f__[*Eubacterium*]_coprostanoligenes_group was positively correlated with isobutyric, valeric, and isovaleric acid proportions (*p* < 0.05). norank_f__UCG-010 was negatively correlated with the acetic acid proportion and positively correlated with isobutyric and isovaleric acid proportions (*p* < 0.05). Conversely, Christensenellaceae_R-7_group was negatively correlated with valeric acid, and *Turicibacter* was negatively correlated with both valeric and isovaleric acid proportions (*p* < 0.05). No significant correlations were detected between TVFA and the dominant genera (*p* > 0.05).

## 4. Discussion

In this study, CS was partially replaced with TS at different inclusion levels (up to 50%) while the forage-to-concentrate ratio and TMR feeding regimen remained unchanged. Under such conditions, changing the forage source primarily affects the nutrient profile of the silages rather than the overall feeding system. Compared with CS, TS contained higher CP, EE, NDF, and ADF, whereas TS generally contained less starch than CS [3,6,7]. Therefore, the replacement strategy was more likely to modify the composition and structure of utilizable substrates, particularly the balance between readily fermentable carbohydrates and structural carbohydrates, than to alter feeding management per se [12,13]. Such shifts in substrate profile can influence nutrient digestion, metabolizable nutrient supply, and lactational responses [26,27,28]. Milk yield reflects the integrated outcome of ruminal and hindgut fermentation, nutrient digestion, post-absorptive metabolism, and mammary nutrient partitioning. Accordingly, the lack of a treatment effect on milk yield suggests that replacing CS with TS up to 50% did not materially alter the overall nutritional support for lactation under the present mid-lactation conditions [28]. This is consistent with previous studies reporting that partial substitution of CS with TS in early- or mid-lactation cows did not compromise milk yield [7]. In this study, a significant effect of time was observed for milk yield, which likely reflected normal temporal variation associated with advancing lactation stage and/or environmental influences rather than a sustained dietary effect. Milk composition further indicated that forage substitution did not disrupt the overall nutritional balance. MUN, which results from the diffusion of blood urea into milk and is widely used to evaluate protein nutrition and nitrogen-use efficiency in dairy cows, did not differ among treatments and remained within a reasonable physiological range [29]. SCC reflects mammary inflammatory status and is closely related to milk quality losses and nutrient repartitioning toward immune responses; therefore, consistently low SCC values help exclude udder-health stress as a confounding factor when evaluating dietary effects on milk traits [30]. In the present study, SCC values (9.86–16.89 × 10^3^ cells/mL) were far below the commonly cited threshold for healthy, uninflamed quarters (<1.0 × 10^5^ cells/mL) [31], suggesting good overall mammary health and no detectable increase in mastitis risk due to TS inclusion. Taken together, these findings indicate that, when diet formulation and feeding management are held constant, partial replacement of CS with TS may be more readily reflected in hindgut fermentation and microbial ecology than in immediate changes in macroscopic production traits; however, longer-term studies are needed to determine whether such microbial shifts translate into broader physiological or health outcomes [11,12,13].

The fecal microbiota represents the terminal gut ecosystem, where undigested residues can undergo secondary fermentation and microbial metabolites may modulate intestinal immunity and nutrient utilization, thereby influencing overall health and performance [32]. Compared with the CON group, the TS50 group showed higher Ace, Shannon, and Chao1 indices, indicating increased community richness and evenness. Principal coordinate analysis (PCoA) and ANOSIM further demonstrated significant between-group differences in community structure, and the TS25 group clustered between CON and TS50, suggesting a dose-dependent shift in the microbial community along the replacement gradient. This gradual displacement pattern is consistent with adaptive restructuring of gut microbial consortia in response to changes in feed substrate supply [12,13]. Similar trends have been reported in related replacement studies, graded substitution of corn grain with sugar beet pulp increased fecal microbial diversity and enriched fiber-associated taxa (e.g., Fibrobacter), accompanied by changes in hindgut fermentation profiles [33], and inclusion of high-NDF by-products altered bacterial communities and fermentation characteristics in both rumen and feces [34]. Therefore, the stepwise shift observed here likely reflects changes in carbohydrate composition and structure caused by TS substitution. At the phylum level, only Fibrobacterota increased significantly in TS25, whereas other dominant phyla remained relatively stable, suggesting that the overall community framework was largely maintained. Fibrobacterota includes efficient degraders of structural carbohydrates within ruminant gastrointestinal ecosystems [35], and its enrichment may indicate a more favorable niche for fiber degradation at moderate replacement levels. In contrast to the relative stability at higher taxonomic ranks, genus-level profiling and LEfSe revealed pronounced redistribution of multiple functionally relevant taxa, indicating that treatment effects were expressed primarily through shifts in specific microbial guilds. The [*Eubacterium*]_coprostanoligenes_group has been associated with conversion of cholesterol to coprostanol, reflecting adaptation in intestinal sterol and lipid metabolism [36]. Given the higher EE content of TS in the present study, the increased relative abundance of this group in replacement treatments may represent an adaptive response to altered lipid inputs and sterol metabolism; however, this hypothesis requires validation via direct coprostanol quantification and metabolomics. *Romboutsia*, for which genomic and functional evidence supports utilization of diverse carbohydrates (monosaccharides and oligosaccharides) and flexible fermentative metabolism [37], decreased in the replacement groups. *Turicibacter* (an anaerobic Gram-positive genus originally described from mammalian samples) [38] also decreased concurrently, which may collectively suggest a shift in hindgut substrate availability from readily fermentable carbohydrates toward more structural, fiber-derived residues. In addition, the uncultured lineage *UCG-010* was specifically enriched in TS50, supporting the notion that higher replacement levels can promote functional remodeling of the terminal gut community, although its ecological role remains to be clarified.

Spearman correlation analysis further revealed associations between dominant fecal genera and milk traits/metabolic indicators, as well as fecal VFA molar proportions, helping to interpret the TS-induced hindgut remodeling, despite unchanged milk yield, 4% FCM, and milk composition. In nitrogen metabolism, *Monoglobus* and norank_f__p-2534-18B5_gut_group were positively correlated with MUN, whereas *Turicibacter* and *Romboutsia* were negatively correlated. Because MUN reflects diffusion of blood urea into milk and is widely used to evaluate protein nutrition and nitrogen use efficiency [29], these opposite relationships suggest that diet-sensitive hindgut guilds may covary with urea turnover and N partitioning. This aligns with the higher CP and fiber in TS compared with CS and with LEfSe showing *Monoglobus* enrichment in TS25 and *Romboutsia*/*Turicibacter* enrichment in CON. At the fermentation level, TS50 did not alter TVFA but reduced acetate proportion while increasing isobutyric, isovaleric, and valeric acids, indicating a shift in end-product distribution; correspondingly, taxa favored by TS (*UCG-010* and [*Eubacterium*]_coprostanoligenes_group) were positively correlated with branched-chain/minor VFAs, and *UCG-010* was negatively correlated with acetate. Regarding energy status, *Prevotellaceae_UCG-003* correlated positively with Acetone and BHBA, and Candidatus*_Saccharimonas* with Acetone. Milk ketone bodies track systemic ketone status and are practical indicators of negative energy balance and hyperketonemia [39,40,41]; thus, these taxa may reflect subtle between-cow variation in energy balance, even though acetone and BHBA remained low and unaffected by TS. *Alistipes* was positively correlated with lactose percentage, supporting a plausible microbial–metabolic link because lactose synthesis depends on glucose supply and ruminant glucose is largely derived from hepatic gluconeogenesis, with propionate as a major precursor [42,43]. Negative correlations of *Clostridium* with milk fat/total solids and of *Treponema* with milk protein suggest individual-level covariation in milk solids partitioning, but the lack of treatment effects on milk composition and the low SCC values indicative of good udder health [30,31] imply no adverse impact of TS on milk quality. Overall, these correlations complement the diversity and VFA results and support the view that TS maintains production while functionally reshaping the hindgut ecosystem under changing substrate supply [32,33,34], warranting causal tests with blood metabolites and multi-omics. However, because the present trial lasted only 60 d, the results should be interpreted as short-term responses; longer-term studies are needed to determine whether these microbial shifts translate into broader health outcomes, including immune status and reproductive performance, and whether similar responses would be maintained under wider farm-scale conditions.

## 5. Conclusions

In summary, partially replacing CS with TS at up to 50% of the corn silage fraction in the diets of mid-lactation Holstein cows did not affect milk yield, 4% FCM, and milk composition over a 60-day period. However, the 50% replacement diet significantly increased the richness and diversity of the fecal microbiota and induced a distinct shift in its overall community structure. Specific bacterial taxa, including fibrolytic and metabolically relevant genera, were modulated by TS inclusion. Additionally, changes in fecal VFA profiles suggest that TS inclusion may influence hindgut fermentation and metabolic pathways, further supporting its potential as a complementary forage to corn silage for lactating dairy cows. Future studies should evaluate longer feeding periods, replicated-pen designs, broader replacement gradients, and additional health and reproductive endpoints before extrapolating these findings to long-term farm adoption.

## Figures and Tables

**Table 1:** Chemical composition of corn silage and triticale silage (DM basis, %).

Items ^1^	Silage ^2^		SEM ^3^	*p*-Value
	CS	TS		
DM, %	29.85	27.20	0.342	<0.001
CP, % of DM	8.08	11.22	0.314	<0.001
EE, % of DM	2.78	3.82	0.042	0.002
NDF, % of DM	47.00	50.35	0.423	0.032
ADF, % of DM	31.68	33.93	0.382	0.037
Ash, % of DM	7.09	8.03	0.043	0.004

^1^ DM: dry matter; CP: crude protein; EE: ether extract; NDF: neutral detergent fiber; ADF: acid detergent fiber; Ash: total ash content. ^2^ CS: corn silage; TS: triticale silage. ^3^ SEM: standard error of the mean.

**Table 2:** Ingredient and nutrient composition of experimental diets (DM basis, %).

Items	CON	TS25	TS50
Ingredients, % of DM			
Corn silage	41.16	30.87	20.58
Triticale silage	0.00	10.29	20.58
Alfalfa silage	7.89	7.89	7.89
Alfalfa hay	7.82	7.82	7.82
Brewers’ grains	3.29	3.29	3.29
Brewers’ yeast	3.18	3.18	3.18
Corn	18.92	18.92	18.92
Wheat	4.02	4.02	4.02
Soybean meal	5.59	5.59	5.59
Corn distillers dried grains with solubles	2.46	2.46	2.46
Premix ^1^	1.00	1.00	1.00
Limestone powder	0.65	0.65	0.65
Dicalcium phosphate	0.10	0.10	0.10
Na_2_HPO_4_	0.05	0.05	0.05
NaCl	0.24	0.24	0.24
Wheat bran	2.19	2.19	2.19
Corn germ meal	0.30	0.30	0.30
Beet pulp	0.26	0.26	0.26
NaHCO_3_	0.88	0.88	0.88
Total	100.00	100.00	100.00
Nutrient components, % of DM			
Crude protein (CP)	14.31	14.60	14.89
Ether extract (EE)	3.19	3.28	3.40
Crude ash (Ash)	8.02	8.12	8.21
Neutral detergent fiber (NDF)	35.1	35.52	35.93
Acid detergent fiber (ADF)	23.08	23.37	23.65
Ca	0.91	0.88	0.85
Phosphorus	0.37	0.37	0.37

CON, control; TS25, 25% triticale silage replacement; TS50, 50% triticale silage replacement. DM, dry matter. ^1^ Premix provided per kg of diet: Zn, 11,000 mg; Mn, 3500 mg; Fe, 1500 mg; Cu, 1000 mg; I, 200 mg; Se, 80 mg; Co, 50 mg; vitamin D, 185,000 IU; vitamin A, 580,000 IU; vitamin E, 3500 IU.

**Table 3:** Effects of partially replacing corn silage with triticale silage on milk yield and 4% FCM of lactating Holstein cows.

Items ^1^	Treatment ^2^			SEM	*p*-Value ^3^				
	CON	TS25	TS50		Trt	Time	Trt × Time	L	Q
Milk yield, kg/d	29.66	29.41	29.78	0.85	0.883	<0.001	0.320	0.914	0.790
4% FCM, kg/d	30.26	30.01	30.73	0.87	0.582	<0.001	0.020	0.688	0.683

Values are least-squares means (LSMeans) with SEM (standard error of the mean). Milk yield and 4% FCM were analyzed at the cow level (*n* = 24 cows per treatment). ^1^ Milk yield (kg/d): daily milk production per cow; 4% FCM (kg/d): 4% fat-corrected milk yield. ^2^ CON: control diet with corn silage as the primary forage; TS25: diet in which 25% of corn silage (DM basis) was replaced by triticale silage; TS50: diet in which 50% of corn silage (DM basis) was replaced by triticale silage. ^3^ Trt: treatment; Time: day (repeated measure); Trt × Time: treatment-by-day interaction; L: linear contrast; Q: quadratic contrast.

**Table 4:** Effects of partially replacing corn silage with triticale silage on milk quality of lactating Holstein cows.

Items ^1^	Treatment ^2^			SEM	*p*-Value ^3^				
	CON	TS25	TS50		Trt	Time	Trt × Time	L	Q
Fat, %	4.10	4.27	4.16	0.31	0.894	0.927	0.766	0.680	0.951
Protein, %	3.35	3.44	3.35	0.10	0.157	0.027	0.249	0.235	0.064
Tots, %	13.31	12.41	13.18	0.88	0.283	0.468	0.224	0.933	0.115
MUN, mg/dL	11.99	12.76	11.81	1.86	0.631	0.034	0.356	0.344	0.873
SCC, ×10^3^ cells/mL	14.15	16.89	9.86	5.59	0.077	0.562	0.232	0.105	0.108
Lactose, %	4.92	4.98	4.91	0.11	0.849	NA	NA	0.920	0.577
Acetone, mmol/L	0.06	0.05	0.05	0.02	0.932	NA	NA	0.741	0.875
BHBA, mmol/L	0.06	0.05	0.04	0.01	0.453	NA	NA	0.228	0.730

Values are least-squares means (LSMeans) with SEM (standard error of the mean). Milk traits were analyzed at the cow level (*n* = 24 cows per treatment). Lactose, Acetone, and BHBA were measured only once at the end of the trial; therefore, Time and Trt × Time effects were not applicable and are shown as NA. ^1^ Fat: milk fat percentage; Protein: milk protein percentage; Tots: total solids percentage; MUN: milk urea nitrogen (mg/dL), reflecting protein metabolism status; SCC: somatic cell count (×10^3^ cells/mL), log10-transformed prior to analysis, with values presented as back-transformed means; Lactose: lactose percentage; Acetone: acetone (mmol/L); BHBA: β-hydroxybutyrate (mmol/L). ^2^ CON: control diet with corn silage as the primary forage; TS25: diet in which 25% of corn silage (DM basis) was replaced by triticale silage; TS50: diet in which 50% of corn silage (DM basis) was replaced by triticale silage. ^3^ Trt: treatment; Time: sampling time; Trt × Time: treatment-by-day interaction; L: linear contrast; Q: quadratic contrast.

## Data Availability

The data will be made available from the corresponding author upon reasonable request.

## References

[1] Xu C., Cao Z., Wu H., Wang B., Niu D., Zheng M., Jiang D., Xiao J., Yang F., Ni K., Guo X. (2022). The Status of the Forage Utilization Industry in China. Research Progress on Forage Production, Processing and Utilization in China.

[2] Bach A., Joulie I., Chevaux E., Elcoso G., Ragués J. (2021). Milk performance and rumen microbiome of dairy cows as affected by the inclusion of corn silage or corn shredlage in a total mixed ration. Animal.

[3] Harper M.T., Oh J., Giallongo F., Roth G.W., Hristov A.N. (2017). Inclusion of wheat and triticale silage in the diet of lactating dairy cows. J. Dairy Sci..

[4] Plaizier J.C., Krause D.O., Gozho G.N., McBride B.W. (2008). Subacute ruminal acidosis in dairy cows: The physiological causes, incidence and consequences. Vet. J..

[5] Zebeli Q., Aschenbach J.R., Tafaj M., Boguhn J., Ametaj B.N., Drochner W. (2012). Invited review: Role of physically effective fiber and estimation of dietary fiber adequacy in high-producing dairy cattle. J. Dairy Sci..

[6] Ayalew H., Kumssa T.T., Butler T.J., Ma X.F. (2018). Triticale improvement for forage and cover crop uses in the Southern Great Plains of the United States. Front. Plant Sci..

[7] Giuliotti L., Benvenuti M.N., Martini A., Accorsi P.A., Lotti C., Cappucci A., Conte G. (2022). Assessment of blood and productive parameters in mid-lactation dairy cows fed different diets: Replacement of corn silage with triticale silage. Arch. Anim. Breed..

[8] Iommelli P., Zicarelli F., Amato R., Musco N., Sarubbi F., Bailoni L., Lombardi P., Di Bennardo F., Infascelli F., Tudisco R. (2024). The Effects of Hemp Hay (*Cannabis sativa* L.) in the Diets of Grazing Goats on Milk Production and Fatty Acid Profile. Animals.

[9] Amato R., Oteri M., Chiofalo B., Zicarelli F., Musco N., Sarubbi F., Pacifico S., Formato M., Lombardi P., Di Bennardo F. (2024). Diet Supplementation with Hemp (*Cannabis sativa* L.) Inflorescences: Effects on Quanti-Qualitative Milk Yield and Fatty Acid Profile on Grazing Dairy Goats. Vet. Q..

[10] Hosseinian F.S., Mazza G. (2009). Triticale bran and straw: Potential new sources of phenolic acids, proanthocyanidins, and lignans. J. Funct. Foods.

[11] Henderson G., Cox F., Ganesh S., Jonker A., Young W., Janssen P.H., Global Rumen Census Collaborators (2015). Rumen microbial community composition varies with diet and host, but a core microbiome is found across a wide geographical range. Sci. Rep..

[12] Ricci S., Rivera-Chacon R., Petri R.M., Reisinger N., Zebeli Q., Castillo-Lopez E. (2022). Progressive microbial adaptation of the bovine rumen and hindgut in response to increasing amounts of starch with or without phytogenic feed additive. Front. Microbiol..

[13] Rivera-Chacon R., Pacífico C., Ricci S., Petri R.M., Reisinger N., Zebeli Q., Castillo-Lopez E. (2024). Prolonged feeding of high-concentrate diet remodels the hindgut microbiome and modulates nutrient degradation in the rumen and the total gastrointestinal tract of cows. J. Dairy Sci..

[14] National Research Council (2001). Nutrient Requirements of Dairy Cattle: Seventh Revised Edition, 2001.

[15] AOAC International (2002). Official Methods of Analysis.

[16] Van Soest P.J., Robertson J.B., Lewis B.A. (1991). Methods for dietary fiber, neutral detergent fiber, and nonstarch polysaccharides in relation to animal nutrition. J. Dairy Sci..

[17] Gaines W.L., Davidson F.A. (1923). Relation Between Percentage Fat Content and Yield of Milk: Correction of Milk Yield for Fat Content.

[18] Chen S., Zhou Y., Chen Y., Gu J. (2018). fastp: An ultra-fast all-in-one FASTQ preprocessor. Bioinformatics.

[19] Magoč T., Salzberg S.L. (2011). FLASH: Fast length adjustment of short reads to improve genome assemblies. Bioinformatics.

[20] Edgar R.C. (2010). Search and clustering orders of magnitude faster than BLAST. Bioinformatics.

[21] Edgar R.C. (2013). UPARSE: Highly accurate OTU sequences from microbial amplicon reads. Nat. Methods.

[22] Wang Q., Garrity G.M., Tiedje J.M., Cole J.R. (2007). Naive Bayesian classifier for rapid assignment of rRNA sequences into the new bacterial taxonomy. Appl. Environ. Microbiol..

[23] Quast C., Pruesse E., Yilmaz P., Gerken J., Schweer T., Yarza P., Peplies J., Glöckner F.O. (2013). The SILVA ribosomal RNA gene database project: Improved data processing and web-based tools. Nucleic Acids Res..

[24] Schloss P.D., Westcott S.L., Ryabin T., Hall J.R., Hartmann M., Hollister E.B., Lesniewski R.A., Oakley B.B., Parks D.H., Robinson C.J. (2009). Introducing mothur: Open-source, platform-independent, community-supported software for describing and comparing microbial communities. Appl. Environ. Microbiol..

[25] Segata N., Izard J., Waldron L., Gevers D., Miropolsky L., Garrett W.S., Huttenhower C. (2011). Metagenomic biomarker discovery and explanation. Genome Biol..

[26] Allen M.S. (1996). Physical constraints on voluntary intake of forages by ruminants. J. Anim. Sci..

[27] Fessenden S.W., Ross D.A., Block E., Van Amburgh M.E. (2020). Comparison of milk production, intake, and total-tract nutrient digestion in lactating dairy cattle fed diets containing either wheat middlings and urea, commercial fermentation by-product, or rumen-protected soybean meal. J. Dairy Sci..

[28] Gross J.J. (2022). Limiting factors for milk production in dairy cows: Perspectives from physiology and nutrition. J. Anim. Sci..

[29] Zhao X., Zang C., Zhao S., Zheng N., Zhang Y., Wang J. (2025). Assessing milk urea nitrogen as an indicator of protein nutrition and nitrogen utilization efficiency: A meta-analysis. J. Dairy Sci..

[30] Stocco G., Summer A., Cipolat-Gotet C., Zanini L., Vairani D., Dadousis C., Zecconi A. (2020). Differential somatic cell count as a novel indicator of milk quality in dairy cows. Animals.

[31] National Mastitis Council (2001). Guidelines on Normal and Abnormal Raw Milk Based on Somatic Cell Counts and Signs of Clinical Mastitis.

[32] Wang D., Tang G., Zhao L., Wang M., Chen L., Zhao C., Liang Z., Chen J., Cao Y., Yao J. (2023). Potential roles of the rectum keystone microbiota in modulating the microbial community and growth performance in goat model. J. Anim. Sci. Biotechnol..

[33] Petri R.M., Münnich M., Zebeli Q., Klevenhusen F. (2019). Graded replacement of corn grain with molassed sugar beet pulp modulates the fecal microbial community and hindgut fermentation profile in lactating dairy cows. J. Dairy Sci..

[34] Lyu J., Yang Z., Wang E., Liu G., Wang Y., Wang W., Li S. (2022). Possibility of using by-products with high NDF content to alter the fecal short chain fatty acid profiles, bacterial community, and digestibility of lactating dairy cows. Microorganisms.

[35] Flint H.J., Bayer E.A., Rincon M.T., Lamed R., White B.A. (2008). Polysaccharide utilization by gut bacteria: Potential for new insights from genomic analysis. Nat. Rev. Microbiol..

[36] Gérard P. (2014). Metabolism of cholesterol and bile acids by the gut microbiota. Pathogens.

[37] Gerritsen J., Hornung B., Renckens B., van Hijum S.A.F.T., Martins dos Santos V.A.P., Rijkers G.T., Schaap P.J., de Vos W.M., Smidt H. (2017). Genomic and functional analysis of Romboutsia ilealis CRIBT reveals adaptation to the small intestine. PeerJ.

[38] Bosshard P.P., Zbinden R., Altwegg M. (2002). Turicibacter sanguinis gen. nov., sp. nov., a novel anaerobic, Gram-positive bacterium. Int. J. Syst. Evol. Microbiol..

[39] Benedet A., Manuelian C.L., Zidi A., Penasa M., De Marchi M. (2019). Invited review: β-hydroxybutyrate concentration in blood and milk and its associations with dairy cow health and performance. Animal.

[40] Tatone E.H., Gordon J.L., Hubbs J., LeBlanc S.J., DeVries T.J., Duffield T.F. (2016). A systematic review and meta-analysis of the diagnostic accuracy of point-of-care tests for the detection of hyperketonemia in dairy cows. Prev. Vet. Med..

[41] Tatone E.H., Duffield T.F., LeBlanc S.J., DeVries T.J., Gordon J.L. (2017). Investigating the within-herd prevalence and risk factors for ketosis in dairy cattle in Ontario as diagnosed by the test-day concentration of β-hydroxybutyrate in milk. J. Dairy Sci..

[42] Rigout S., Hurtaud C., Lemosquet S., Bach A., Rulquin H. (2003). Lactational effect of propionic acid and duodenal glucose in cows. J. Dairy Sci..

[43] Aschenbach J.R., Kristensen N.B., Donkin S.S., Hammon H.M., Penner G.B. (2010). Gluconeogenesis in dairy cows: The secret of making sweet milk from sour dough. IUBMB Life.

